# Functionally conserved enhancers with divergent sequences in distant vertebrates

**DOI:** 10.1186/s12864-015-2070-7

**Published:** 2015-10-30

**Authors:** Song Yang, Nir Oksenberg, Sachiko Takayama, Seok-Jin Heo, Alexander Poliakov, Nadav Ahituv, Inna Dubchak, Dario Boffelli

**Affiliations:** Genomics Division, Lawrence Berkeley National Laboratory, Berkeley, CA 94720 USA; Department of Bioengineering and Therapeutic Sciences, University of California San Francisco, San Francisco, CA 94158 USA; Institute for Human Genetics, University of California San Francisco, San Francisco, CA 94158 USA; Children’s Hospital Oakland Research Institute, Oakland, CA 94609 USA; US Department of Energy Joint Genome Institute, 2800 Mitchell Drive, Walnut Creek, CA 94598 USA

## Abstract

**Background:**

To examine the contributions of sequence and function conservation in the evolution of enhancers, we systematically identified enhancers whose sequences are not conserved among distant groups of vertebrate species, but have homologous function and are likely to be derived from a common ancestral sequence. Our approach combined comparative genomics and epigenomics to identify potential enhancer sequences in the genomes of three groups of distantly related vertebrate species.

**Results:**

We searched for sequences that were conserved within groups of closely related species but not between groups of more distant species, and were associated with an epigenetic mark of enhancer activity. To facilitate inferring orthology between non-conserved sequences, we limited our search to introns whose orthology could be unambiguously established by mapping the bracketing exons. We show that a subset of these non-conserved but syntenic sequences from the mouse and zebrafish genomes have homologous functions in a zebrafish transgenic enhancer assay. The conserved expression patterns driven by these enhancers are probably associated with short transcription factor-binding motifs present in the divergent sequences.

**Conclusions:**

We have identified numerous potential enhancers with divergent sequences but a conserved function. These results indicate that selection on function, rather than sequence, may be a common mode of enhancer evolution; evidence for selection at the sequence level is not a necessary criterion to define a gene regulatory element.

**Electronic supplementary material:**

The online version of this article (doi:10.1186/s12864-015-2070-7) contains supplementary material, which is available to authorized users.

## Background

In eukaryotes, the expression state of a given gene is controlled by one or more enhancers. Enhancers are not spatially restricted to the region proximal to the gene’s transcription start site but can be located anywhere, including in introns or exons of distal genes. The spatial and temporal pattern of activity of an enhancer is controlled by the type of epigenetic modifications attached to it, and by the combinatorial binding of transcription factors to specific binding sites in the enhancer’s sequence [1].

The sequences of many enhancers are under selection in vertebrates [2, 3]. Based on this observation, genomic scans for noncoding regions characterized by low rates of sequence divergence among different species have been used to identify evolutionarily conserved enhancers. This strategy has been most successfully applied to highly distant species (e.g., human and fish) [4], but it has also been applied within closely related species (e.g., primates [5]). However, conservation of a noncoding sequence is not highly correlated with enhancer activity. Some highly conserved noncoding sequences are not associated with any obvious functional activity [6], and many experimentally verified enhancers are weakly or not conserved between distant species [7–9], indicating that conservation of a specific sequence, to the extent that it can be detected by phylogenetic methods [10], is not a requirement for enhancer activity.

Stabilizing selection can explain why the function of some enhancers is conserved in related species while their sequences are not. Ludwig *et al*. showed that the sequence of the *even-skipped* stripe 2 enhancer was not conserved in two *Drosophila* species because of transcription factor binding site (TFBS) turnover, but that the activity pattern of the enhancer was restrained to the same stripe and developmental stage in both species [11]. Similar results have been noted for other enhancers in *Drosophila melanogaster* [12–14], and for the *endo16* promoter in sea urchins [15]. In vertebrates, syntenic sequences of *RET* in human and pufferfish lacked any sequence homology but exhibited similar gene regulatory activities in a zebrafish enhancer assay [16]. In these studies, compensatory mutations and the rearrangement of TFBSs were an important source of sequence divergence.

It has been argued that only sequences that are shown to be under some type of selection can be claimed to be functional with any degree of confidence [17]. This criterion will miss enhancers such as those described in the previous paragraph: the ancestral function of these enhancers has been conserved but their sequences have become unalignable [18]. The observation that the function of an enhancer, rather than its sequence, may be the main factor under selection suggests that related species have the potential to share many enhancers of similar function but divergent sequence; this type of enhancers would be invisible to genomic approaches based on sequence conservation.

Here we present a strategy for the systematic identification of evolutionarily related enhancers that have a conserved activity but divergent sequences. We searched for sequences that were conserved within groups of closely related species but not alignable between groups of more distant species, and that were associated with an epigenetic mark of enhancer activity. To facilitate inferring orthology between non-alignable sequences, we focused on enhancers found within orthologous introns of three groups of distant vertebrate species. We show that a subset of these non-conserved enhancers have homologous activities in a zebrafish transgenic enhancer assay. The conserved expression patterns driven by these enhancers are likely to be associated with short transcription factor-binding motifs present in the divergent sequences. We have identified numerous potential enhancers with divergent sequences but a conserved activity; these results indicate that selection on function, rather than sequence, may be a common mode of enhancer evolution. These enhancers might help shed light on the factors driving the evolution of gene regulatory sequences and developing models of enhancer evolution.

## Results

### Identification of evolutionarily conserved regions

**Fig. 1:**
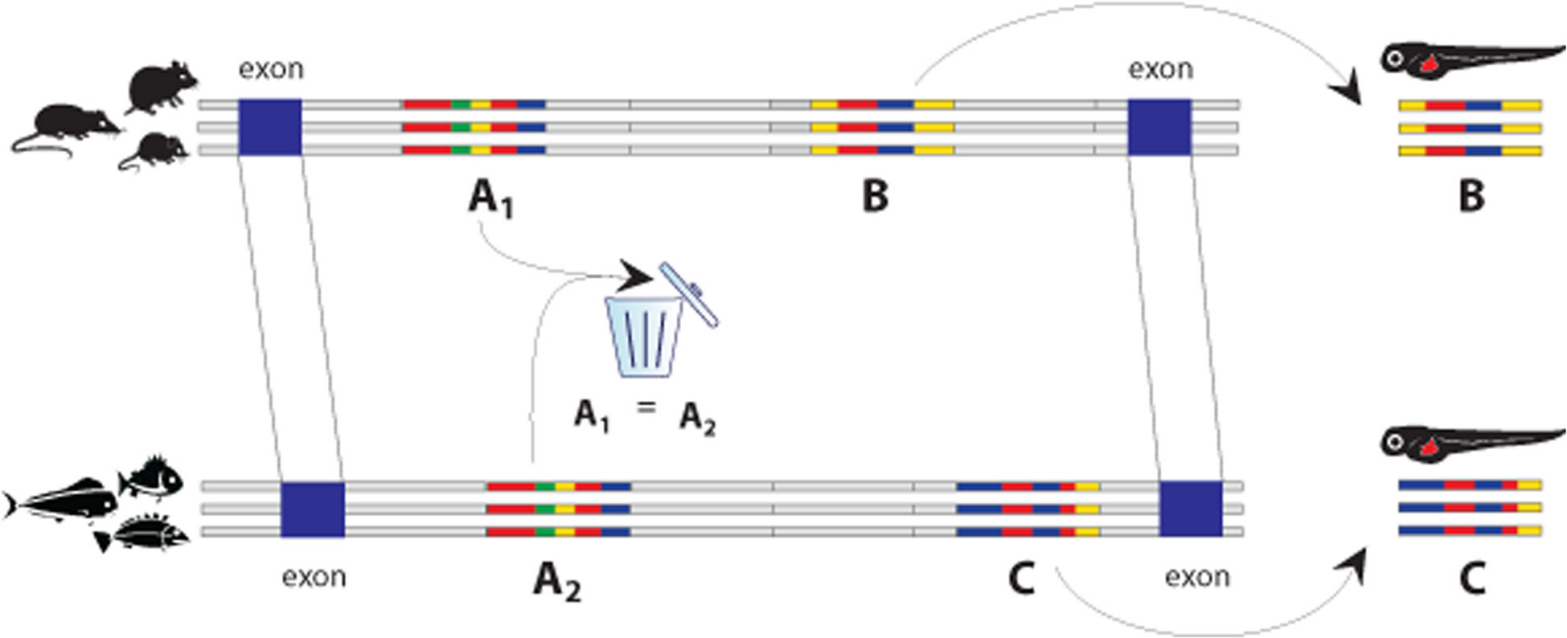
Scheme of the analysis of orthologous introns bracketed by the orthologous exons in the rodent/rabbit and fish evolutionary groups of genomes. Conserved sequences (*A*1 and *B*, and *A*2 and *C* in multiple alignments of the groups 1 and 2 respectively) are compared, and highly similar *A*1 and *A*2 removed from the analysis. Sequences *B* and *C* are selected for experimental validation

The goal of this study was to identify enhancers that have similar spatiotemporal patterns of expression in related species, and that are likely to be derived from a common ancestral enhancer but have lost detectable sequence conservation. We developed a procedure to identify sequences in three distantly related species (mouse, chicken and zebrafish) that are (1) potential regulatory sequences, (2) orthologous, and (3) not conserved between the species. Since lack of sequence conservation prevents establishing orthology by alignment, we limited our search to intronic sequences whose orthology was established by aligning their bracketing exons. We used a combination of comparative genomics and epigenomics to search for potential enhancers within this set of orthologous introns, following the steps illustrated in Fig. 1 and described in Methods.

As the first step of the procedure outlined above, we selected three groups of species (Table 1) based on the following criteria: (a) each species has a high-quality genome assembly; (b) the species within each group are characterized by a level of sequence divergence that is suitable for the identification of candidate enhancers by comparative genomics [19]; (c) the groups are distantly related to each other: their genomes have low sequence similarity but are alignable at the exon level. We generated multiple alignments of the genomes of the species within each group using VISTA [20, 21], and identified exons that are orthologous between all the three groups. We used the RefSeq annotations [22] of a representative species from each group and the human genome (chosen because it has the most complete annotation) as common reference. Since the RefSeq annotations of chicken and zebrafish are incomplete, we identified additional orthologous exon pairs by projecting the human annotation on the other species’ genome and confirming the orthology of the projected exons by inspecting the sequence alignment of the projected exons in the representative species. We then identified pairs of adjacent orthologous exons (within a genome), and used these pairs to define sets of orthologous introns between each of the species (see Fig. 1); the results of this analysis are shown in Table 2. Finally, we identified a subset of 17,921 orthologous introns shared by mouse, chicken and zebrafish (Table 2). Only these introns were used in the subsequent analyses.

**Table 1:** Genome assemblies used in this study

	Species	Genome assembly
Rodent + Rabbit	Mus musculus (Mouse)	Jul. 2007 (mm9)
	Rattus norvegicus (Rat)	Nov. 2004 (rn4)
	Cavia porcellus (Guinea pig)	Feb. 2008 (cavPor3)
	Oryctolagus cuniculus (Rabbit)	Apr. 2009 (oryCun2)
Bird	Gallus gallus (Chicken)	May 2006 (galGal3)
	Taeniopygia guttata (Zebra finch)	Jul. 2008 (taeGut1)
	Meleagris gallopavo (Turkey)	Dec. 2009 (melGal1)
Fish	Danio rerio (Zebrafish)	Jul. 2010 (danRer7)
	Takifugu rubripes (Fugu)	Oct. 2004 (fr2)
	Tetraodon nigroviridis (Tetraodon)	Mar. 2010 (tetNig2)
	Gasterosteus aculeatus (Stickleback)	Feb. 2006 (gasAcu1)
	Oryzias latipes (Medaka)	Oct. 2005 (oryLat2)

Reference genomes for each of the four groups are listed first

**Table 2:** Number of orthologous introns between pairs of species and groups of three species

	Number of annotated orthologous introns	Projected introns	Total number
Human-Mouse	127,176	-	127,176
Human-Chicken	22,815	59,465	82,280
Human-Zebrafish	27,337	24,999	52,336
Mouse-Chicken-Zebrafish			17,921

We used sequence conservation to identify candidate enhancer sequences within the orthologous intron set defined in Table 2. We obtained ECRs genome-wide by running Phastcons on the multiple whole-genome alignments of the members of the rodent, bird, and fish groups (Table 3, first row), and selected the subset that maps to the set of 17,921 orthologous introns described in the previous paragraph (Table 3, second row). In order to retain only ECRs that are not conserved between groups, we discarded the ECRs that were conserved between at least two of the three groups of species (Table 3, third row – the process is illustrated in Fig. 1). Since the last filter leaves some introns devoid of ECRs in at least one of the groups, we retained the orthologous introns that contained one or more ECR in each group (Table 3, fourth row). These ECRs showed a univocal correspondence in only a few introns across the three groups. Most orthologous introns contained a different number of ECRs in each group; due to the lack of sequence conservation between these regions, we could not establish a precise one-to-one orthology between ECRs in orthologous rodent-bird-fish introns; we will refer to these ECRs as “syntenic ECRs”.

**Table 3:** Rodent-bird-fish comparison

	Rodent	Bird	Fish
Intra-group conserved regions genome-wide	350,003	178,982	394,456
Conserved noncoding regions (ECR) in orthologous introns	6,390	4,079	5,044
Intra-group intronic ECRs not conserved in other groups	4,814	2,904	3,625
ECRs located in introns with ECRs in all groups of species	1,415	1,220	1,447
overlapping with H3K4me1 but not H3K4Me3	728	*(n/a)*	273

To obtain further support for potential enhancer activity, we determined which of the ECRs identified by the comparative genomic procedure described above overlapped with H3K4me1 annotations but not with H3K4me3 annotations. H3K4me1 is a histone modification mark associated with distal regulatory regions [23], while H3K4me3 is associated with promoter activity [23]; genome-wide data are available for both histone marks in the mouse [24] and zebrafish [25], but are not available for bird species. The lack of histone mark data for bird reduces the power of our analysis to identify; concordant for H3K4me1 binding from three distant species would improve the confidence that our computational pipeline identified functional enhancers. The sequences of 728 rodent ECRs and 273 fish ECRs that overlapped with H3K4me1 annotations but not H3K4me3 annotations were considered the candidate enhancers (Table 3 – 47 of the rodent regions and 61 of the fish regions overlapped with both marks). The proportion of ECRs associated with H3K4me1 may be lower for fish because histone modification data were obtained only from whole embryos at 24 h post fertilization (hpf), rather than from multiple tissues and developmental time points as for the mouse.

### Experimental validation of computationally predicted enhancers

The candidate enhancers located in orthologous segments of the genome may be derived from a common ancestral sequence and may thus have maintained a similar spatial and/or temporal pattern of enhancer activity. To test this possibility, we selected 22 zebrafish and mouse ECRs among the syntenic rodent-bird-fish intronic ECRs from Table 3 and subjected them to an enhancer assay in zebrafish (Additional file 1: Figure S1). We selected ECRs located in introns that contained up to two ECRs (to facilitate the implication of a univocal correspondence between syntenic ECRs) and within genes expressed during early development (the transgenic assay is carried out at 24 and 48 hpf).

**Fig. 2:**
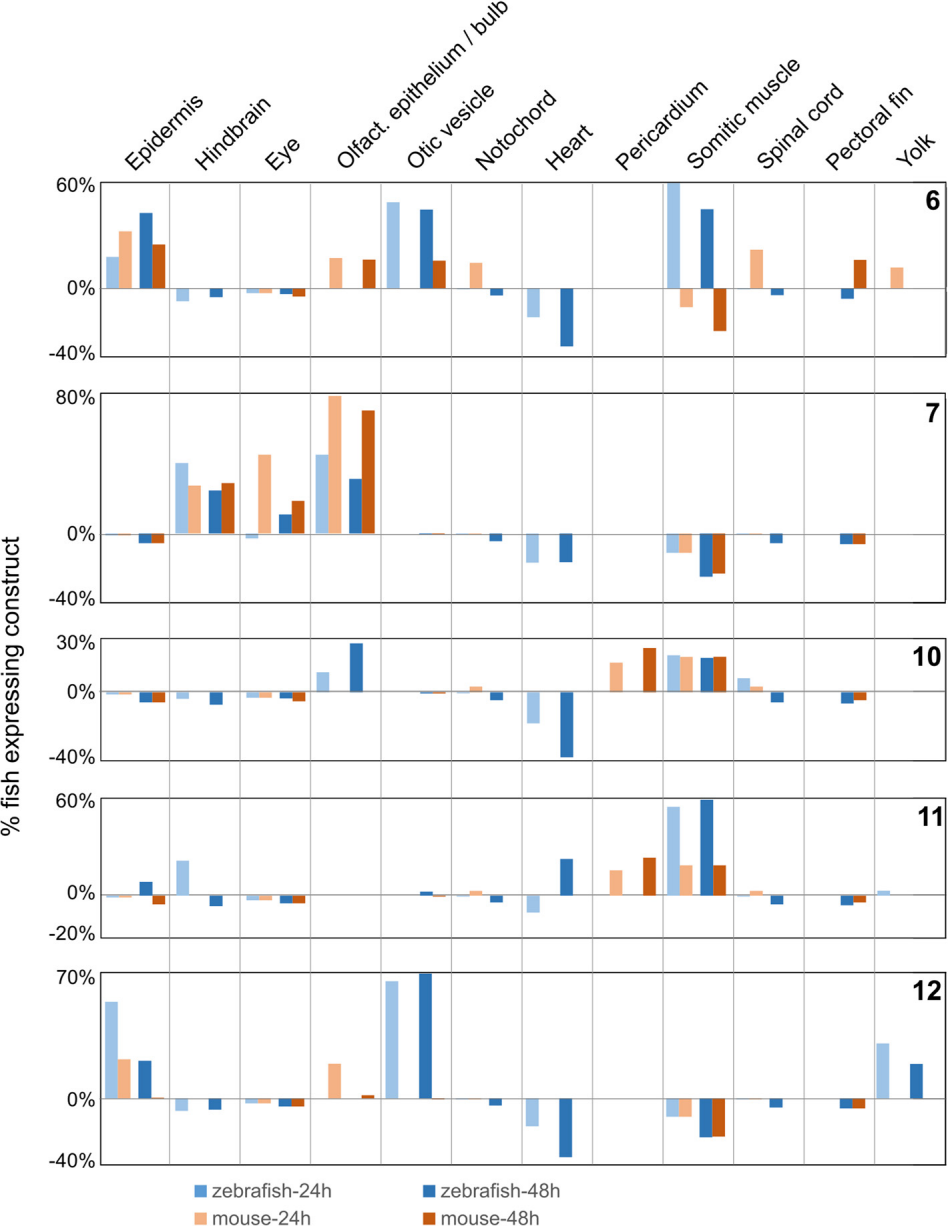
Expression data in for positive zebrafish (blue) and mouse (brown) ECR constructs in transgenic zebrafish. The x-axis shows tissues with positive expression in at least one construct; the y-axis shows the number of fish expressing a construct in a given tissue (minus the number of fish with ectopic expression – see Additional file 2: Table S1 for details), reported as the proportion of fish alive at 24 hpf (light color) and 48 hpf (dark color). The identity of each construct is shown in the top right corner of each chart. The expression of the mouse ECR syntenic to zebrafish ECRs 10 and 11 is reported in both charts

**Fig. 3:**
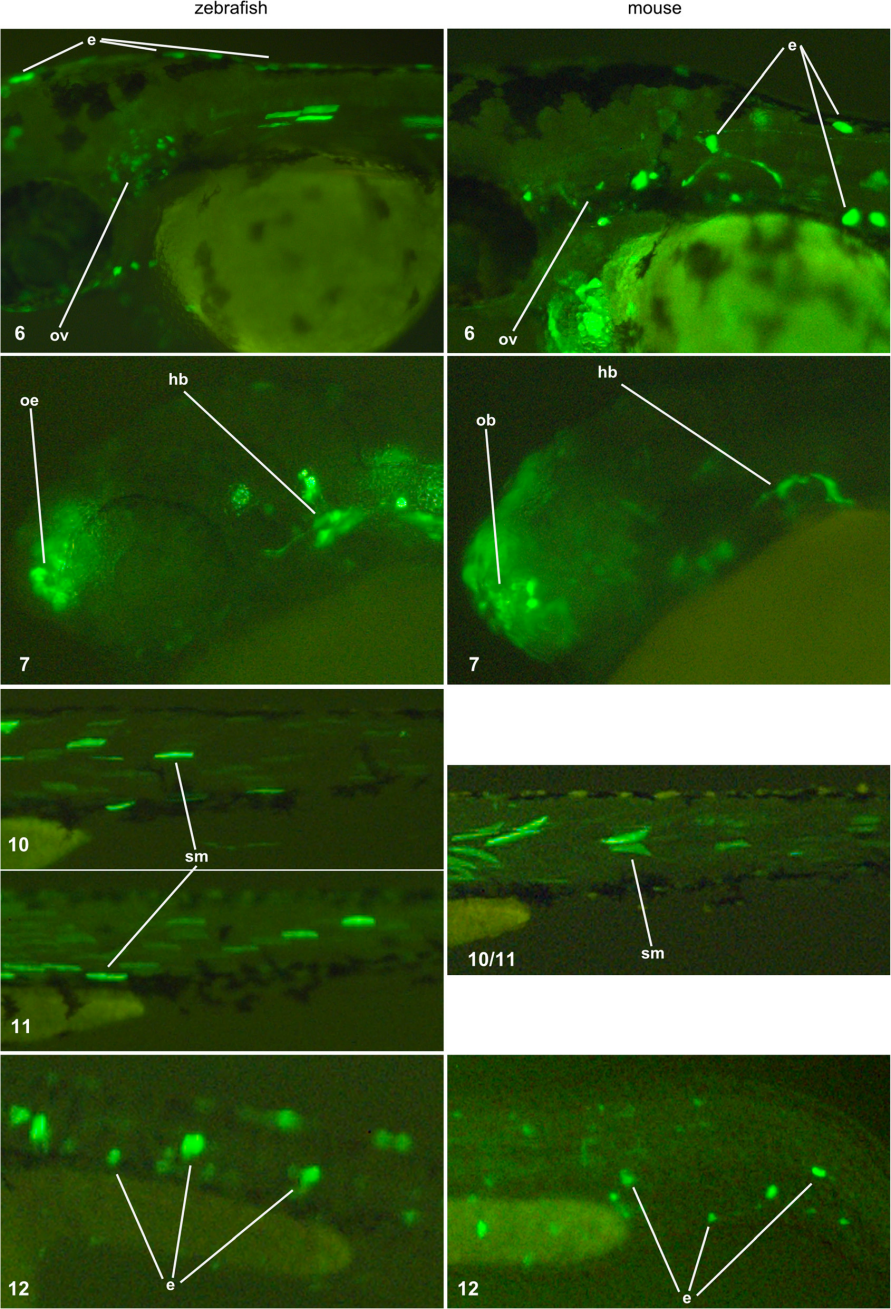
Expression patterns driven by zebrafish and mouse syntenic ECRs with homologous activity in a zebrafish transgenic enhancer assay. ECRs are identified by the number at the bottom left of each panel, their genomic coordinates are in Table 4. Expression patterns were recorded at 24 hpf (ECR 7) or 48 hpf (ECRs 6, 10, 11, and 12) and detected by GFP expression (green). Labels indicate tissues in which homologous mouse and zebrafish sequences show consistent patterns of GFP expression (see Experimental Procedures for the definition of consistent pattern of expression). ECR 6: constructs from both species drive strong expression in several epidermal cells; the zebrafish construct also drives expression in the otic vesicle , weak staining at the periphery of the vesicle is visible with the mouse construct (see also Additional file 1: Figure S1). ECR 7: the mouse construct drives expression in the olfactory bulb, but expression by the zebrafish construct is restricted to the olfactory epithelium (the sensory component of the olfactory bulb – see also Additional file 1: Figure S1); constructs from both species drive expression in large neurons in the hindbrain of. ECR 10 and 11: one ECR is present in the mouse, and two ECRs are present in the orthologous zebrafish intron; constructs from both species drive clear expression in somitic muscle cells. ECR12: constructs from both species drive clear expression in several epidermal cells. Abbreviations – e: epidermis; ov: otic vesicle; oe: olfactory epithelium; ob: olfactory bulb; hb: hindbrain; sm: somitic muscle

We first tested the 22 zebrafish ECRs. Each ECR was PCR-amplified from the zebrafish genome, cloned into a reporter vector containing an E1b minimal promoter upstream of GFP, and injected into zebrafish embryos to detect enhancer activity [26]. Of the 22 zebrafish regions tested, 13 (59 %) showed consistent enhancer activity in multiple tissues at either 24 or 48 hpf (Table 4 and Figs. 2 and 3; the complete dataset showing the proportion of live fish with expression driven by a ECR in a given tissue is shown in Additional file 2: Table S1; Additional file 3: Table S2 lists constructs that yielded negative results).

**Table 4:** Zebrafish and mouse predicted enhancers subjected to experimental validation

ECR	Zebrafish (Zv9 July 2010)		Mouse (mm9) July 2007)	
	Expression	Coordinates	Expression	Coordinates
1	Spinal cord (48)	chr6:20,797,116-20,798,106	negative	chr5:107,545,775–107,546,750
2	Epidermis (24,48)	chr6:43,538,446–43,539,094	negative	chr6:98,881,656–98,882,625
			negative	chr6:98,883,956–98,884,889
*3*	*Tail bud (24), eye (48), heart (48)*	*chr9:11,657,708–11,658,704*	*not tested*	
4	Notochord (24,48)	chr19:22,713,465–22,714,026	negative	chr18:80,876,694–80,877,675
			negative	chr18:80,876,778–80,877,739
*5*	*Epidermis (24,48)*	*chr19:41,272,614–41,273,241*	*not tested*	
**6**	**Epidermis** (24,48), **Otic Vesicle** (24,48), Somitic muscle (24,48)	chr2:39,790,777–39,791,782	**Epidermis** (24,48), Notochord (24), Olfactory epithelium (24,48), Spinal cord (24), **Otic Vesicle** (48), Pectoral fin (48)	chr1:77,387,974–77,389,004
**7**	**Hindbrain** (24,48), **Olfactory epithelium** (24,48)	chr2:39,792,312–39,793,241	Eye (24,48), **Olfactory bulb** (24,48), **Hindbrain** (24,48)	chr1:77,390,034–77,391,031
8	Somitic muscle (24,48), Olfactory epithelium (48), Hindbrain (48)	chr2:39,837,096–39,838,009	negative	chr1:77,503,689–77,504,676
			negative	chr1:77,506,344–77,507,445
9	Spinal cord (24,48)	chr4:16,470,975–16,471,819	Midbrain (24), Yolk (24)	chr10:87,004,979–87,005,964
			Somitic Muscle (24,48), Skin under yolk (24,48), Phar. Arches (48), Pectoral fin (48)	chr10:87,008,830–87,009,792
**10**	**Somitic muscle** (24,48), Olfactory epithelium (48)	chr9:22,365,926–22,366,799	**Somitic Muscle** (24,48), Pericardium (24,48)	chr14:58,234,894–58,235,874
**11**	Hindbrain (24), **Somitic muscle** (24,48), Heart (48)	chr9:22,368,460–22,369,405		
**12**	**Epidermis** (24,48), Otic vesicle (24,48), Yolk (24,48)	chr14:41,645,428–41,646,386	**Epidermis** (24), Olfactory epithelium (24)	chrX:130,162,955–130,163,923
13	Hindbrain (24), Olfactory epithelium (24,48), Line under eye (24,48), Spinal cord (48)	chr20:21,970,078–21,970,911	Spinal cord (24)	chr12:72,999,373–73,000,279

Tissues in bold (in ECR 6, 7, 10, 11, 12) show consistent enhancer activity between the mouse and zebrafish syntenic enhancers at the indicated time point (24 or 48 hpf). Each construct was injected in at least 100 embryos; the proportion of fish alive and expressing in a given tissue is shown in Additional file 2: Table S1

We then asked if the mouse ECRs from introns orthologous to those containing the 13 positive zebrafish enhancers also have enhancer activity, and if their activity patterns overlap with the patterns observed in the corresponding zebrafish enhancers. We PCR-amplified 14 mouse ECRs corresponding to 11 of the zebrafish enhancers (Table 4): two zebrafish enhancers (regions 10 and 11) were syntenic to a single mouse ECR; each of four zebrafish ECRs (regions 2, 4, 8, 9) were syntenic to two mouse ECRs; and the mouse ECRs syntenic the zebrafish ECRs in regions 3 and 5 were not tested because of difficulties in obtaining the PCR products. The mouse ECRs were cloned in the same zebrafish reporter vector used for the zebrafish constructs, and injected into zebrafish (the proportion of live fish showing expression driven by a ECR in a given tissue is shown in Additional file 2: Table S1). We used zebrafish to test mouse ECRs in order to compare the expression patterns obtained from the mouse and zebrafish ECRs using the same assay system; also, zebrafish has been successfully used to test mammalian enhancer sequences [16, 27]. Seven mouse ECRs (syntenic to the zebrafish ECRs 1, 2, 4, 8, Table 4) did not show enhancer activity at either 24 or 48 hpf. Three mouse ECRs (syntenic to zebrafish ECRs 9 and 13 – Table 4) showed enhancer activity in the zebrafish assay but in tissues or at a time point different from those observed in their syntenic zebrafish ECRs. Finally, four mouse ECRs (syntenic to zebrafish ECRs 6, 7, 10, 11, 12 – Table 4) showed spatial and temporal enhancer activity patterns that overlapped with the patterns observed in their corresponding zebrafish ECRs (Figs. 2 and 3).

All ECRs with consistent spatio-temporal expression patterns also showed expression in tissues that were detected in constructs from one but not the other species (Table 4). Also, consistent spatial patterns were overlapping but not always identical. ECR 6 showed expression in epithelial cells and the otic vesicle, but in the mouse construct expression was limited to the periphery of the otic vesicle while it is visible throughout the otic vesicle in the zebrafish construct. ECR 7 showed expression in hindbrain neurons, but the zebrafish and mouse constructs were detected in different neurons; the mouse ECR 7 construct also drives diffuse expression in the olfactory bulb, but expression form the zebrafish construct is restricted to the olfactory epithelium, which is the sensory component of the olfactory bulb. Zebrafish ECRs 10 and 11 were syntenic to a single mouse ECR; they both drove expression in multiple tissue, with somitic muscle being the only common one; somitic muscle is also consistent with the expression driven by the syntenic mouse construct. ECR 12 showed strong and consistent expression in epithelial cells. These subtle differences in spatial expression patterns could reflect a degree of subfunctionalization of these enhancers, or the inability of the zebrafish expression system to read with complete accuracy regulatory information carried in mouse sequence.

### Functionally homologous enhancers share transcription factor binding motifs

The common expression patterns observed in the sets of syntenic enhancers suggest that they share functional elements, but their lack of sequence conservation prevents the use of standard sequence-based methods for the identification of such elements. We used BLAST to identify short sequence alignments with a perfect match of at least 7 nucleotides in the zebrafish, mouse, and human sequences of the five sets of syntenic enhancers defined in Table 4. (BLAST allowed us to focus on short identical sequences for a stringent first-pass search of shared functional elements – it is likely that a less stringent search using a motif-finding algorithm may reveal additional motifs.) The five ECR contained different numbers of perfect match n-mers (n ≥ 7): ECR6, 29 n-mers; ECR7, 23 n-mers; ECR10, 19 n-mers; ECR11, 14 n-mers; ECR12, 40 n-mers. The probability that a set of three identical heptamers is identical by chance is 0.28, that is, 4–11 n-mers are expected to be identical by chance – the functionality of these motifs relies on ultimate functional verification, but these data provide guidance to the location of the likely functional motifs.

**Fig. 4:**
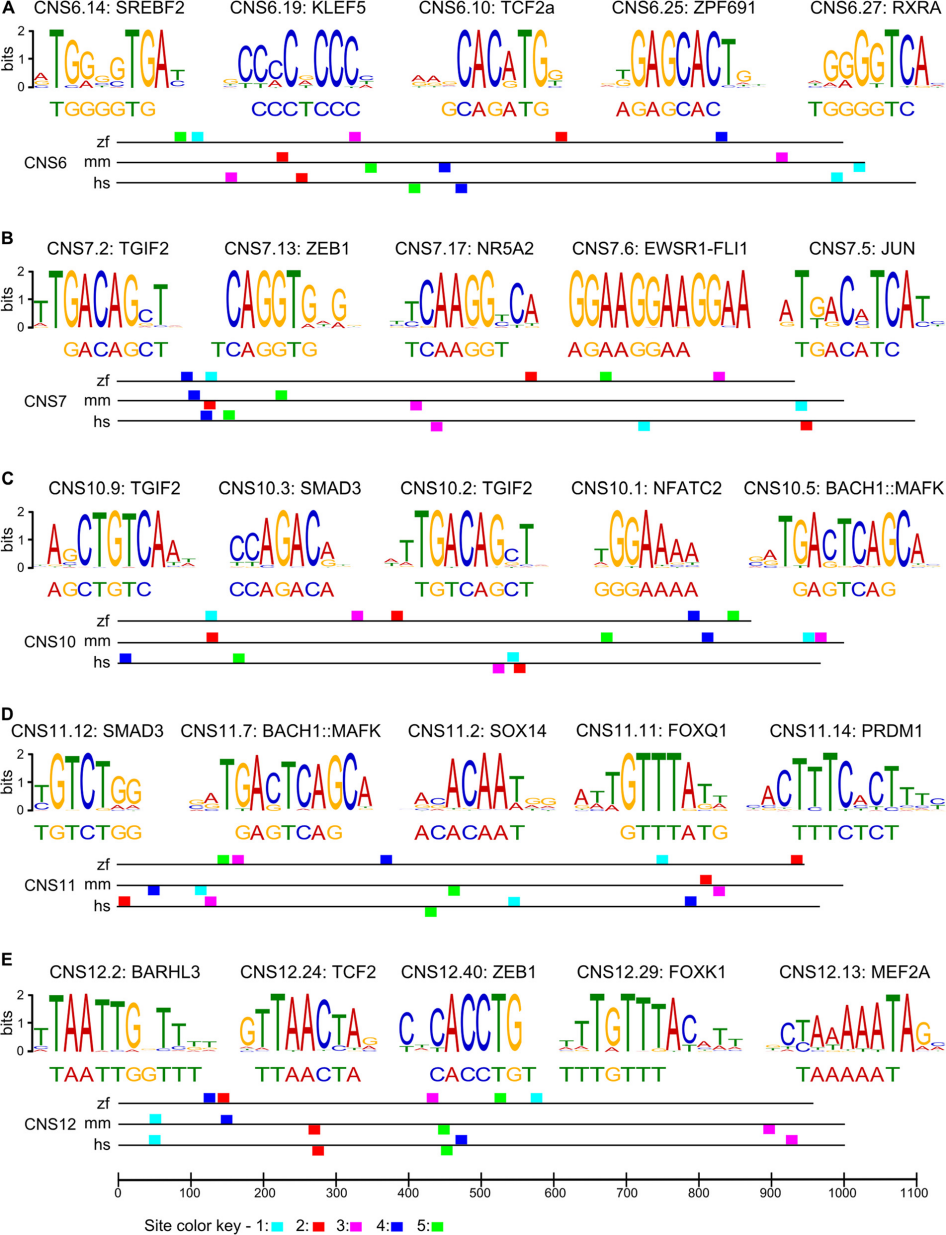
Transcription factor binding sites detected in the five syntenic ECRs. **a-e**: each panel shows the five motifs with most significant E-value in the given ECR, and the sequence of the n-mer, identical in the zebrafish, mouse, and human sequence of the ECR, from which the motif was identified. The name of the transcription factor binding to a motif is shown above the motif, and the sequence of the motif is shown for the strand on which the n-mer was found. The arrangement of the motifs along the zebrafish (zf), mouse (mm), and human (hs) sequences is shown at the bottom of each panel (the scale, in base pairs, is the same for all panels and shown only for panel E). Motifs are color-coded according the legend at the bottom of the figure; motifs shown above and below the reference line are identified on the forward and reverse strands, respectively

We identified TFBS in the sets of perfect-match n-mers by searching the Jaspar [28] and Uniprobe [29] databases using TomTom [30]. Several n-mer sets contained TFBS with E ≤ 0.1 (Fig. 4 and Additional file 4: Table S3). The arrangement of these TFBS along the sequence of a ECR differs among zebrafish, mouse and human (Fig. 4), consistent with the lack of sequence conservation noted at these ECRs. Most of the TFBS are specific to a single ECR, but it is interesting to note that zebrafish ECR10 and ECR11, which are syntenic to a single ECR in the mouse and drive the same expression pattern in the reporter assay (Fig. 3), share two TFBSs with highly significant E-value (SMAD3 and BACH1::MAFK).

## Discussion

We described the systematic identification and validation of functionally syntenic enhancers with divergent sequences in three groups of vertebrate species - rodent, bird and fish. We have devised a comparative genomic strategy to identify enhancers that are likely to be derived from the same ancestral sequence but do not share sequence conservation. We identified 5 sets of syntenic and functionally homologous zebrafish and mouse ECRs: these enhancers were able to drive overlapping spatiotemporal patterns of expression in a zebrafish reporter construct, but lacked detectable sequence conservation.

This functional homology could be derived from stabilizing selection on extant sequences derived from the same ancestral sequence, or from convergent evolution of enhancers derived from ancestrally unrelated sequences. Our data does not allow ruling out the second possibility, but we think that it is unlikely: convergent enhancers may evolve anywhere in the proximity of their target genes and would not be expected to be found systematically within the same introns. Another possibility is that the lack of conservation that we observed at the functionally homologous enhancers is due to incomplete genome assemblies or to the algorithms we used for sequence alignment and the identification of conservation. More complete genome assemblies, or improved and more sensitive algorithms, might detect sequence conservation at these enhancers; while we cannot rule out this possibility, our results were obtained with the best assemblies and computational tools available at the time. The lack of observed sequence homology prevents a direct determination of orthology of the enhancers we found in the orthologous introns (intron orthology can be assigned unambiguously through the alignments of their bracketing exons), but our finding of functional homology between syntenic enhancers suggests that we were able to systematically identify enhancers whose sequences are not conserved among distant groups of vertebrate species, but are likely to be derived from a common ancestral sequence under the action of stabilizing selection. Testing of larger numbers of orthologous intronic enhancers may provide further support for this hypothesis.

What drives the homologous expression patterns of these enhancers? The most obvious hypothesis is that the orthologous enhancers, in spite of their lack of sequence similarity, share common short, functional blocks that are arranged in a different linear order in mouse and zebrafish, in a manner similar to that previously observed in Drosophila and sea urchin enhancers [11, 12, 15]. Our analysis revealed several transcription factor-binding motifs present in the zebrafish, mouse and human sequences of the syntenic enhancers. These motifs are arranged in a different order within the syntenic zebrafish, mouse, and human sequences of a given ECR, consistent with the notion that order of TFBSs can rearrange within an enhancer while maintaining the enhancer’s function. Functional validation of these binding sites will require substantial functional dissection, which could be possible with massively parallel reporter assays [31]. We note that zebrafish ECRs 10 and 12, which are syntenic to the same mouse ECR and drive the same reporter expression pattern, share the binding sites for SMAD3 and BACH1::MAFK; both transcription factors belong to the TFG- β signaling pathway and are involved in cell proliferation and differentiation [32, 33] and might be responsible for the shared enhancer activity of ECRs 10 and 12.

## Conclusions

Our results indicate that selection on function, rather than sequence, may be a common mode of enhancer evolution and should be used as evidence for functionality of a noncoding sequence. Our method was restricted to introns to facilitate the implication of orthology [18]; it can be further expanded to other noncoding sequences such as intergenic intervals if orthology can be established accurately. This new class of active enhancers can significantly expand the collection of known functional elements in the human genome. Characterization of the functional building blocks of these enhancers and of the patterns of rearrangement that are compatible with the maintenance of enhancer activity will assist in the general understanding of how *cis*-regulatory elements function and evolve.

## Methods

### Computational pipeline for the identification of orthologous but divergent candidate enhancer sequences

Our computational analysis included the following steps (see also Fig. 1):

1. Selection of three groups of related species and generation of multiple alignments of the species within each group. The species are closely related within a group, but are distantly related between the groups (rodent and fish alignments are used in Fig. 1 to illustrate the procedure).
2. Identification of orthologous introns between the reference genomes of each group: these introns are defined by their bracketing orthologous exons (shown in blue in Fig. 1).
3. Identification of Evolutionarily Conserved Regions (ECRs). We used the “within group” multiple alignments from Step I to identify ECRs in each group (Fig. 1: regions marked A_1_ and B are conserved within rodents, and regions A_2_ and C within fish).
4. Removal of ECRs from Step III that were also conserved between groups (regions A_1_ and A_2_ in Fig. 1).
5. Intersection of the ECRs passing Step IV (regions B and C in Fig. 1) with experimental data for mono-methylation of histone H3K4, a hallmark of enhancer activity: ECRs within the intersection are the candidate enhancer sequences. A subset of these sequences was experimentally investigated using a transgenic enhancer assay in zebrafish.

### Step 1. Groups of closely related genomes

Selection of the three groups of closely related species (primates, rodents/rabbit, birds, and fish) was based on the number of available complete genome assemblies and evolutionary distances among the species inside and outside the groups (Table 1). The best-annotated genome in each group was selected as a reference genome.

Genome-wide multiple DNA alignments for each of the three groups of species (Table 1) and pair-wise alignments of the three reference genomes and the human genome hg19 were built with the VISTA pipeline [21], a well-established tool for comparative genomics [34–36].

VISTA pipeline uses an alignment algorithm that efficiently combines global and local alignment techniques [20]. It consists of the following steps: obtaining a map of large blocks of conserved synteny between the two species by applying Shuffle-LAGAN glocal chaining algorithm [37] to local alignments by translated BLAT [38]. Alignment is done by PROLAGAN, a variation of the original Multi-LAGAN program that allows for the alignment of two alignments (profiles) and predicting ancestral contigs using a maximum matching algorithm [20].

### Step 2. Finding orthologous introns among the reference genomes

For our analysis, we considered only introns bracketed by the orthologous exons. We used NCBI Homologene database [39] to find orthologous genes in the four reference species, human, mouse, chicken and zebrafish, and built a collection of exons orthologous in all groups. Since the annotation of the human genome is the most complete, the reference species of each group were first compared with the human genome annotation, and then compared to one another. Matching orthologous exons within orthologous genes annotated by RefSeq [22] in each pair of species were identified and confirmed by pairwise sequence alignments. Since gene annotations in some species (e.g. chicken and zebrafish) are incomplete, additional exon pairs were derived from sequence homology. For example, if a pair of orthologous genes in the human and chicken genomes has only a human RefSeq annotation, the chicken exon annotation was inferred by the chicken/human conservation calculated from the VISTA alignment with default parameters of conservation 70 %/100 bp window. Finally, all orthologous pairs of exons obtained by this analysis were compared to one another to verify orthology across all four groups.

### Step 3. Evolutionarily conserved sequences

Evolutionarily conserved elements in the reference genomes were derived from the intra-group multiple alignments using PhastCons [40], that is based on phylo-HMM, a statistical model of sequence evolution. Neutral model was based on four-fold degenerate sites in coding regions, and phyloFit program was used to estimate all the model parameters (branch lengths and transition rates between nucleotides). Separate neutral models for the X chromosome and autosomes were used. The divergence is generally smaller on the X, and if you use the autosomal model there, you end up with elevated conservation scores. The model for the conserved state was obtained by multiplying all branch lengths by the scaling parameter rho (0 < rho < 1; we used its default value of 0.3). Once a neutral model was built PhastCons run on the alignment produced conservation scores genome-wide. Conserved regions found by Phastcons were inspected for overlap: if two conserved regions were overlapped by more than 90 % (relative to either one of the two sequences), only the longer region was retained along with its conservation score.

### Step 4. Co-located evolutionarily conserved sequences (ECRs)

In order to retain intronic sequences conserved among the species in each of the four groups, but not conserved between the groups, we removed ECRs that are found in orthologous introns as well as in any of the pairwise alignments of reference genomes; a minimum overlap of one base pair was used as a criterion for removal. In addition, to verify that the remaining conserved regions within each group do not have homologs in other groups, we used BLAT to search each ECR against the entire sequences of the reference genomes of the other groups, using minScore = 30, minIdentity = 50. The ECRs passing this filter step are our co-located candidate enhancers in orthologous introns.

### Step 5. Prioritization of syntenic enhancers for functional testing using epigenetic annotations and gene ontology analysis

The best scenario is when each intron in a set of orthologous introns contains a single ECR: in this scenario, orthology can be implicated unambiguously i.e. this ECR can be considered as candidate enhancer region. In other cases, if an orthologous intron contains several intra-group conserved regions, the many-to-many relationship makes it impossible to implicate orthology. However, in most cases an intron containing one ECR in one group of species is orthologous to an intron containing two or more ECRs in the other groups, so the search criteria were extended to intronic regions that contain up to three ECRs in each group. These candidate sequences were further filtered by required overlap with mapped histone modification tags. Specifically we selected ECRs that overlapped with H3K4me1, a mark associated with regulatory elements, and that did not overlap with H3K4me3, a mark associated with promoters. These data are available for both human [24] and zebrafish [25].

Finally, we selected ECRs found in the introns of genes associated with the Gene Ontology terms “developmental process” and “anatomical structure development”. After all these filtering step, 22 regions in 20 orthologous introns were selected for experimental validation.

### Transgenic enhancer assays in zebrafish

PCR was carried out on zebrafish or mouse genomic DNA using primers designed to amplify the candidate sequences and an additional ~200 bp flanking sequences on either side of the target region (Additional file 5: Table S4). PCR primers also contained restriction sites for cloning in the reporter vector (Additional file 5: Table S4).

PCR products were cloned into the E1b-GFP-Tol2 enhancer assay vector that contains an E1b minimal promoter followed by GFP [26], sequence-verified using Sanger sequencing from both ends of the PCR product, and injected into zebrafish embryos following standard procedures [41, 42]. At least 100 embryos per construct were injected with Tol2 mRNA [43] to facilitate genomic integration. GFP expression was annotated at 24 and 48 hpf. Enhancers were considered positive if at least 15 % of all fish surviving to 24 or 48 hpf showed consistent expression pattern after subtracting out percentages of tissue expression in fish injected with the empty enhancer vector. All zebrafish experiments were carried out in accordance with Institutional Animal Care and Use Committee regulations of the University of California at San Francisco, protocol Approval # AN100466-03.

### Identification of conserved transcription factor binding sites

We developed a stepwise procedure for finding short sequences that are conserved in orthologous fish, mouse and human non-coding intervals but cannot be detected by direct phylogenetic methods. We applied the following steps to each of the syntenic enhancers:

1. BLAST the zebrafish and mouse sequences and find all the hits of length n ≥ 7 (this is the length of most TFBS). Sequences containing simple repeats were removed (ECR 7 and 11 contained 77 bp and 86 bp with simple repeats, respectively).
2. Find the corresponding human sequence using a pairwise human-mouse alignment as a guide.
3. BLAST the zebrafish and human sequences and find all the hits of length n ≥ 7.
4. Compare the mouse-fish and the human-fish high score BLAST hits from steps 1 and 3 and find overlapping hits of length n ≥ 7.
5. Search for TFBS in the sets of perfect-match n-mers by searching the Jaspar [28] and Uniprobe [29] databases using TomTom with Euclidean distance [30]. Only TFBS with E-value E ≤ 0.1 are reported. Also, if two TFBS are found at overlapping sequences, only the TFBS with smaller E-value is reported.

The probability of observing conserved motifs by chance is calculated as follows. In the pairwise alignments of the five zebrafish ECRs (ECR 6, 7, 10, 11, 12) with their syntenic mouse and human sequences using BLAST [44], we observed 87–157 pairwise alignments of length ≥ 7 (depending on the specific pairwise alignment). The number of alignments of length 7 expected by chance varied between 53 and 73 (depending on the length of the sequences analyzed – the bitscore of a heptamer is 13.9), so that on average 53 % of the pairwise alignments are expected to occur by chance. From this we estimate that the probability of observing the same alignment by chance in the comparison of the zebrafish-mouse and zebrafish-human alignments is 0.53*0.53 = 0.28 (assuming conservatively that the same chance alignments are present in both sets), that is ~28 % of the sequences of length ≥ 7 observed in the analysis of the syntenic zebrafish-mouse-human ECRs are expected to occur by chance.

## Additional files

Additional file 1: Figure S1. Contains additional views of the zebrafish expression driven by the zebrafish and mouse version of Regions 6 and 7. The circle in region 6 identifies the expression in the Otic Vesicle (ov). The zebrafish construct shows expression throughout the otic vesicle, while the mouse construct shows a ring of GFP expression around the periphery of the OV, behind the eye. Region 7 shows expression in the olfactory epithelium (oe, zebrafish construct) and the olfactory bulb (ob, mouse construct). These are histologically overlapping tissues in the forebrain: olfactory epithelium is the sensory component of the olfactory bulb. (PNG 1687 kb) Additional file 2: Table S1. Number of fish expressing a construct in a given tissue, reported as the proportion of fish alive at the experimental time point. The first worksheet shows the fish counts for the CNSs obtained from the zebrafish genome, and the second worksheet shows the fish counts for the CNSs obtained from the mouse genome. Ectopic expression counts driven by the enhancerless (labeled “empty”) vector are shown in the first row of each worksheet and are subtracted from the counts obtained with the vector with enhancer (labeled “insert”) to obtain the correct counts for each construct (labeled “insert-empty”). (XLSX 41 kb) Additional file 3: Table S2. Zebrafish CNSs that did not drive expression activity in the zebrafish transgenic assay. (XLSX 12 kb) Additional file 4: Table S3. Conserved transcription factor binding motifs in the five zebrafish/mouse syntenic enhancers. Identical n-mers (n ≥ 7) identified in the zebrafish, mouse, and human sequences of the five syntenic CNS were examined for the presence of transcription factor binding motifs; only motifs with E-value E ≤ 0.1 are shown. (XLSX 15 kb) Additional file 5: Table S4. List of primers used in the cloning of the reporter vectors. (XLSX 11 kb)
