# Supplementary figures and images for: Functionally conserved enhancers with divergent sequences in distant vertebrates
Source: BMC Genomics. 2015 Oct 30;16:882. doi: 10.1186/s12864-015-2070-7 (PMC4628251; doi:10.1186/s12864-015-2070-7)

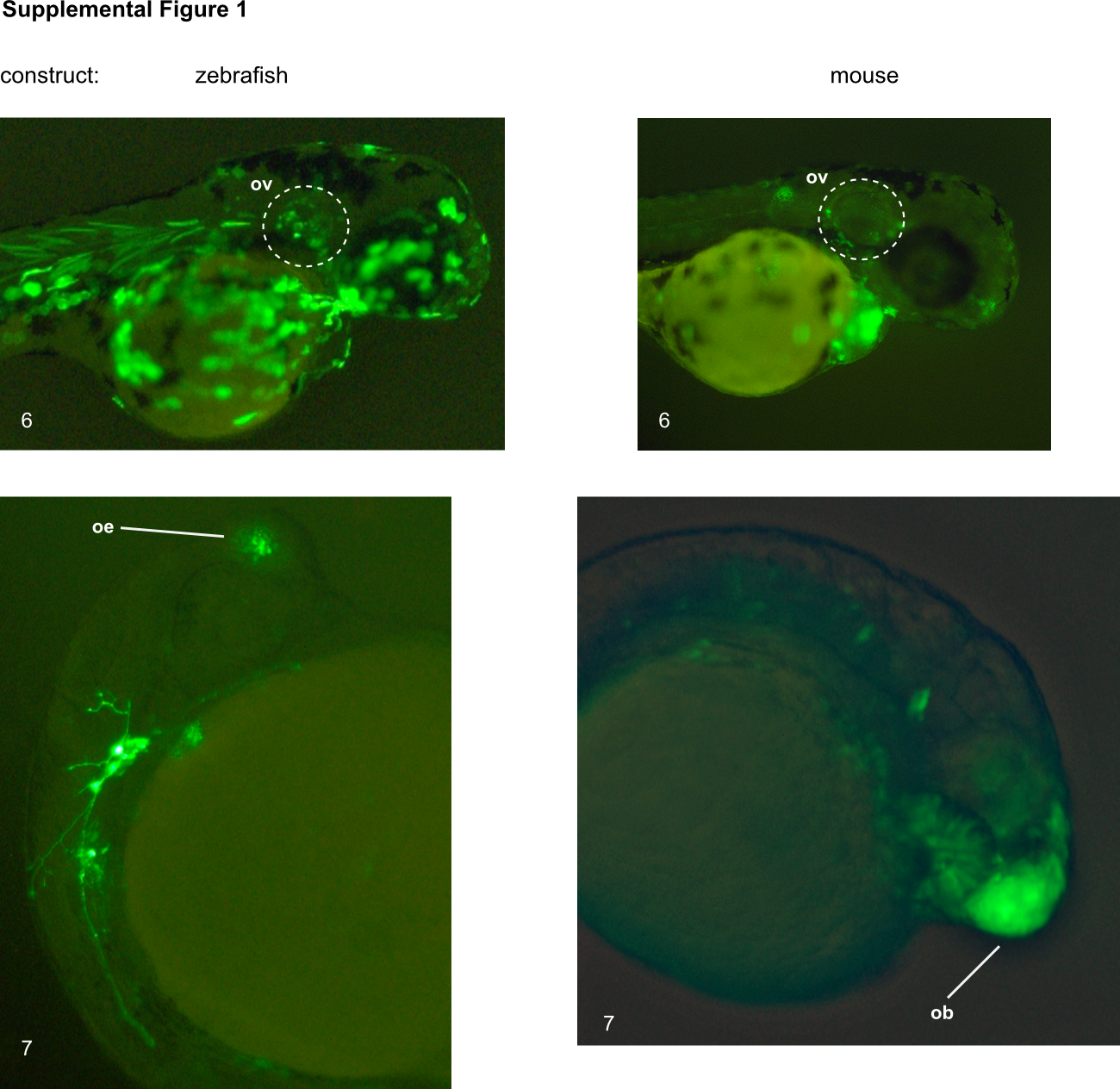

Supplement: Additional file 1: Figure S1. — Contains additional views of the zebrafish expression driven by the zebrafish and mouse version of Regions 6 and 7. The circle in region 6 identifies the expression in the Otic Vesicle (ov). The zebrafish construct shows expression throughout the otic vesicle, while the mouse construct shows a ring of GFP expression around the periphery of the OV, behind the eye. Region 7 shows expression in the olfactory epithelium (oe, zebrafish construct) and the olfactory bulb (ob, mouse construct). These are histologically overlapping tissues in the forebrain: olfactory epithelium is the sensory component of the olfactory bulb. (PNG 1687 kb) [file 12864_2015_2070_MOESM1_ESM.png]
